# SARS-CoV-2 Susceptibility and COVID-19 Mortality Among Older Adults With Cognitive Impairment: Cross-Sectional Analysis From Hospital Records in a Diverse US Metropolitan Area

**DOI:** 10.3389/fneur.2021.692662

**Published:** 2021-07-22

**Authors:** Alan P. Pan, Jennifer Meeks, Thomas Potter, Joseph C. Masdeu, Sudha Seshadri, Matthew Lee Smith, Marcia G. Ory, Farhaan S. Vahidy

**Affiliations:** ^1^Center for Outcomes Research, Houston Methodist, Houston, TX, United States; ^2^Nantz National Alzheimer Center, Stanley H. Appel Department of Neurology, Houston Methodist, Houston, TX, United States; ^3^Weill Cornell Medical College, New York, NY, United States; ^4^Glenn Biggs Institute for Alzheimer's and Neurodegenerative Diseases, University of Texas Health Science Center, San Antonio, TX, United States; ^5^Center for Population Health and Aging, Texas A&M University, College Station, TX, United States; ^6^School of Public Health, Texas A&M Health Science Center, College Station, TX, United States; ^7^Neurological Institute, Houston Methodist, Houston, TX, United States

**Keywords:** cognitive impairment, Alzheimer's disease, dementia, SARS-CoV-2, COVID-19, propensity score matching, patient registries

## Abstract

**Introduction:** Persistent knowledge gaps exist as to the extent that preexisting cognitive impairment is a risk factor for susceptibility to severe acute respiratory syndrome coronavirus 2 (SARS-CoV-2) and mortality from the coronavirus disease 2019 (COVID-19).

**Methods:** We conducted a cross-sectional analysis of adults tested for SARS-CoV-2 at a tertiary healthcare system. Cognitive impairment was identified utilizing diagnosis codes (mild cognitive impairment, Alzheimer's disease, vascular, and other dementias) or cognitive impairment-specific medication use. Propensity score (PS) matched analyses were utilized to report odds ratios (OR) and 95% confidence intervals (CI) for association of cognitive impairment with SARS-CoV-2 susceptibility and COVID-19 mortality.

**Results:** Between March-3rd and December-11th, 2020, 179,979 adults were tested, of whom 21,607 (12.0%) tested positive. We identified 6,364 individuals with preexisting cognitive impairment (mean age: 78.5 years, 56.8% females), among whom 843 (13.2%) tested positive and 139 (19.5%) of those hospitalized died. In the pre-PS matched cohort, cognitive impairment was significantly associated with increased SARS-CoV-2 susceptibility (OR, CI: 1.12, 1.04–1.21) and COVID-19 mortality (OR, CI: 2.54, 2.07–3.12). One-to-one matches were identified for 6,192 of 6,364 (97.3%) individuals with prior cognitive impairment and 687 of 712 (96.5%) hospitalized patients with prior cognitive impairment. In the fully balanced post-matched cohort, preexisting cognitive impairment was significantly associated with higher likelihood of SARS-CoV-2 infection (OR, CI: 1.51, 1.35–1.70); however, cognitive impairment did not confer higher risk of COVID-19 mortality (OR, CI: 0.96, 0.73–1.25).

**Discussion:** To mitigate the effects of healthcare catastrophes such as the COVID-19 pandemic, strategies for targeted prevention and risk-stratified comorbidity management are warranted among the vulnerable sub-population living with cognitive impairment.

## Introduction

The disparate burden of the coronavirus disease 2019 (COVID-19) pandemic across vulnerable sub-populations has been established. Higher vulnerability to the severe acute respiratory syndrome coronavirus 2 (SARS-CoV-2) and poor COVID-19 outcomes are reported to be associated with advanced age and greater comorbidity burden (1, 2); studies have also demonstrated differences in sex (3, 4) and race/ethnicity (5), with respect to SARS-CoV-2 susceptibility and/or disease severity.

Notwithstanding these insights, there is a need to further examine the clinical factors that may underlie the SARS-CoV-2 infection and COVID-19 pathways and help identify high-risk subgroups, particularly among the frail and older population. Although SARS-CoV-2 is primarily a respiratory pathogen, multi-organ dysfunction—including the central nervous system (CNS)—is now widely reported (6, 7). In particular, neurological associations with COVID-19 include a broad range of symptoms and events (e.g., anosmia/ageusia, seizures, delirium, stroke, and cerebrovascular disease) (8). However, there is limited evidence about the role that prior history of cognitive impairment plays in SARS-COV-2 infection and COVID-19 prognosis.

Among the older population, cognitively impaired individuals may be even more susceptible to SARS-CoV-2 infection as well as the overall clinical and social consequences of the COVID-19 pandemic. Although broad narrative overviews highlighting impact and challenges of COVID-19 among patients with Alzheimer's disease and dementia have been authored (9, 10); systematic age and comorbidity adjusted analyses examining the vulnerability to SARS-CoV-2 and outcomes of COVID-19 among individuals with preexisting cognitive impairment are limited, particularly those representing large racial and ethnic diverse cohorts in the United States (US). These insights are crucial for precise risk-stratification and ultimate mitigation of the impacts of current and related pandemics.

We evaluated and quantified the risk of SARS-CoV-2 infection and COVID-19 mortality among individuals with prior history of cognitive impairment who were tested and treated across a large tertiary healthcare system in a Southern metropolitan area. We hypothesized that prior cognitive impairment is independently associated with a higher likelihood of SARS-CoV-2 susceptibility and COVID-19 mortality.

## Methods

### Study Setting and Design

Houston Methodist (HM) is an eight-hospital tertiary healthcare system, which comprises an extensive network of primary care and emergency medicine services, including an accountable care organization across the greater Houston metropolitan in Texas. Following the “strengthening the reporting of observational studies in epidemiology” (STROBE) guidelines, we conducted a cross-sectional secondary data analysis from our Institutional Review Board (IRB) approved (PRO00025445) COVID-19 Surveillance and Outcomes Registry (CURATOR). The detailed rationale and design of CURATOR has been previously reported (11). Briefly, the CURATOR is a validated big-data repository for all individuals who underwent a polymerase chain reaction (PCR) test (regardless of symptom presentation or test result) for presence of SARS-CoV-2 RNA in nasopharyngeal specimens across HM. The CURATOR design allows for longitudinal information capture before and after SARS-CoV-2 testing or COVID-19 hospitalization and readily provides appropriate controls for various analyses. The CURATOR is a robust structured query language data repository with automated extract transform and load procedures directly built interfacing with institutional electronic health records. CURATOR captures a broad range of demographic, medical history, laboratory, medication, treatment, and outcomes variables for all individuals. Given the prevalence of cognitive impairment among younger populations, we included all adult (≥18 years) individuals tested for SARS-CoV-2 at HM between March 3rd and December 11th, 2020. Of note, we restricted our data extraction to SARS-CoV-2 tests performed prior to COVID-19 vaccine rollout at HM on December 15th, 2020.

### Primary Exposure and Outcomes

Utilizing commonly documented International Classification of Diseases, Tenth Revision (ICD-10) diagnosis codes for history of mild cognitive impairment, Alzheimer's disease, vascular and other dementias (F00, F01, F02, F03, F05.1, F10.73, F11.73, F14.73, F16.73, F18.73, F19.73, G30, and G31.84) or history of prior use of cognitive impairment-specific medications (donepezil, rivastigmine, galantamine, and memantine), we identified individuals with cognitive impairment (12).

We analyzed two primary outcomes (i.e., susceptibility to SARS-CoV-2 among tested individuals and in-hospital mortality from COVID-19). Individual and patient-level analyses were conducted for SARS-CoV-2 infection (susceptibility) and in-hospital mortality, respectively. All SARS-CoV-2 PCR tests in CURATOR were flagged and the subset with laboratory-confirmed positive results was identified as having a positive outcome for susceptibility. Among individuals with multiple SARS-CoV-2 PCR tests, individuals were categorized “positive” if they ever had a positive test result and ‘negative’ if all test results were negative. Data from the first positive or negative clinical encounter were included for either category of individuals.

Separately, all COVID-19 hospitalizations in CURATOR were flagged by identifying hospitalization encounters with a primary discharge diagnosis of ICD-10: U07.1, regardless of SARS-CoV-2 testing status. Patients who died during a hospitalization encounter for COVID-19 were flagged for in-hospital mortality and hospitalization data for these encounters were included. Among patients who did not experience in-hospital mortality and who had multiple hospitalization events, data were analyzed from their first COVID-19 hospitalization.

### Other Covariates

Other variables of interest included demographic factors (age, sex, race, ethnicity, and marital status), socio-economic indicators (insurance, ZIP Code abstracted US Census estimates for household income, poverty, and population density), address geocoded neighborhood area deprivation indices (ADI), and an aggregate burden of 17 comorbidities quantified by the Charlson Comorbidity Index (CCI) (13). Encounter-level data (age, residence, and insurance) was queried to abstract the most recent information with respect to testing and hospitalization. Vital signs at admission, laboratory measures (white blood cell count, lymphocytes, platelet count, B-natriuretic peptide, procalcitonin, troponin, aspartate aminotransferase, alanine aminotransferase, total bilirubin, C-reactive protein, ferritin level, D-dimer, creatinine, and venous lactate), medications (hydroxychloroquine, ribavirin, azithromycin, lopinavir/ritonavir, remdesivir, tocilizumab, antithrombotics, anticoagulants, and dexamethasone), hospital course complications (pneumonia, acute respiratory distress syndrome, bronchitis, lower respiratory tract infection, acute renal injury, acute hepatic injury, heart failure, and respiratory failure), and acuity-of-care factors (intensive care and mechanical ventilation) were also included.

### Statistical Analyses

Descriptive statistics (means, standard deviations [SD], interquartile ranges [IQR], and proportions) and group difference testing were performed to report bivariable comparisons. Continuous variables were assessed for deviation from normality using the Shapiro–Wilk test, and non-parametric evaluation (Wilcoxon rank-sum test, Mood's median test) was used for non-normal distributions. Nominal variables were evaluated using Chi-squared tests. Simple logistic regression models were fit to estimate the unadjusted odds and 95% confidence intervals (95% CI) of SARS-CoV-2 susceptibility and COVID-19 mortality associated with cognitive impairment. Propensity score (PS) matching was performed distinctly for each set of susceptibility and mortality analyses to select controls without cognitive impairment matched to cases with cognitive impairment. Individual participant-level PS were calculated as the probability of exposure (preexisting cognitive impairment vs. no cognitive impairment) given a set of covariates by fitting a multivariable logistic regression model. Variables included in the PS model were based on evidence from prior literature for factors that influence susceptibility to SARS-CoV-2 (likelihood of a positive PCR test result from among all tested individuals) and COVID-19 associated mortality (likelihood of in-hospital mortality from among all COVID-19 related hospitalizations); and included socio-demographics (age, sex, race, ethnicity, marital status, insurance, and ADI), comorbidities (CCI score, obesity, diabetes, and hypertension), hospital admission vital signs (systolic and diastolic blood pressure, respiratory rate, temperature, and oxygen saturation), and acuity-of-care factors (utilization of intensive care mechanical ventilation resources). PS-based, nearest neighbor, one-to-one matching was employed and post-matched standardized mean difference (SMD) estimating the average treatment effect on the treated was evaluated in the pre- vs. post-matched samples. Pre- and post-matched odds ratios (OR) and 95% confidence intervals (95% CI) are reported. Conditional independence between prior cognitive impairment and study outcomes (SARS-CoV-2 infection and in-hospital COVID-19 mortality) across sex strata was evaluated using Cochran–Mantel–Haenszel tests. Analyses were performed using R statistical software (The R Foundation; version 3.6.1) and required packages (“MatchIt”; version 4.1.0).

## Results

**Figure 1:**
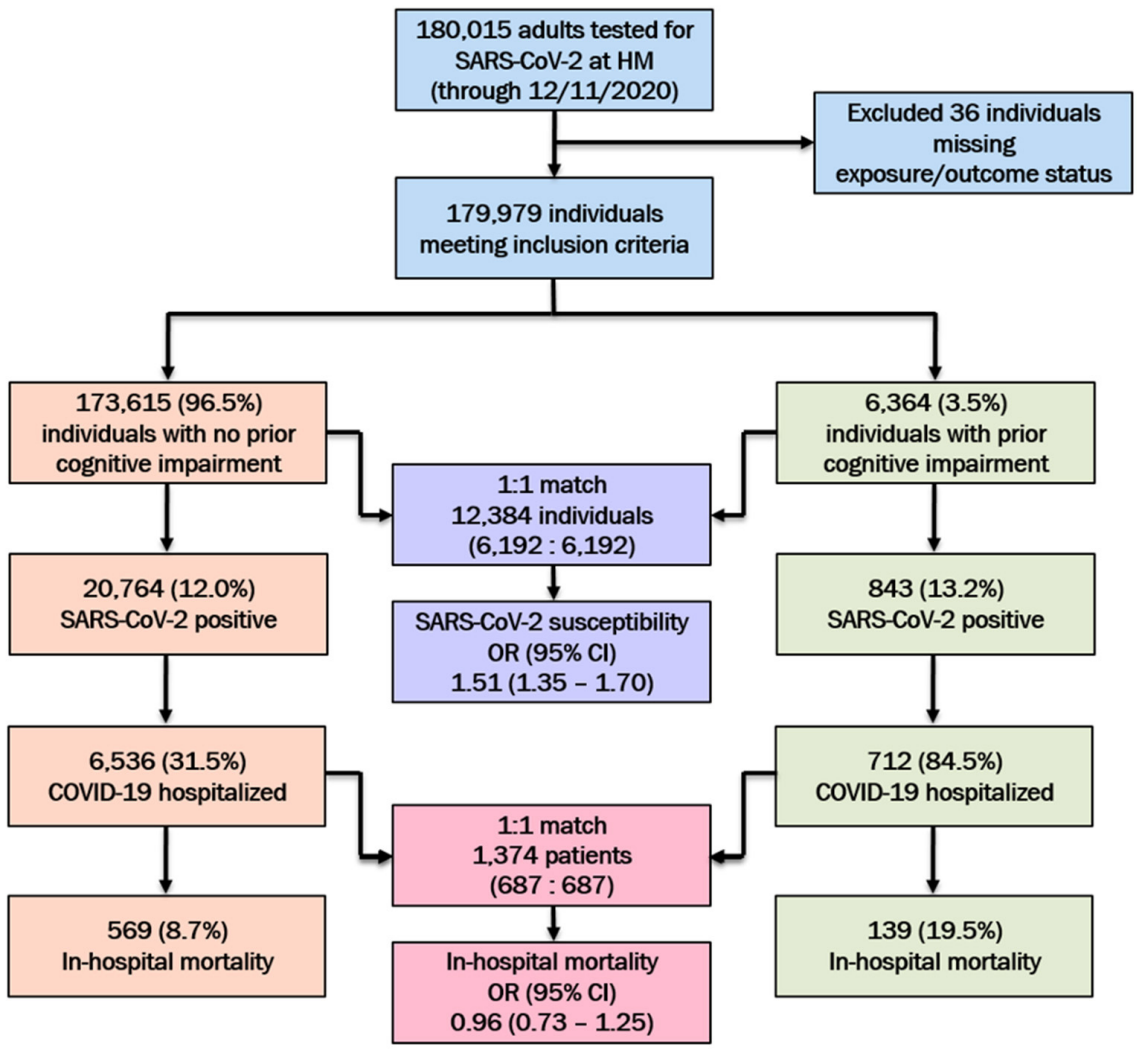
SARS-CoV-2 testing characteristics and hospital outcomes among the study population at Houston Methodist (HM), stratified by preexisting cognitive impairment status. Post-propensity score matched odds ratios (ORs) and 95% confidence intervals (95% CI) for SARS-CoV-2 susceptibility and in-hospital mortality among the cognitively impaired (vs. no cognitive impairment) population presented.

Between March 3rd and December 11th, 2020; a total of 179,979 adults were PCR-tested for SARS-CoV-2, of whom 21,607 (12.0%) tested positive. Among the positive cases, 7,248 (33.5%) were hospitalized, of whom 708 (9.8%) died. The study population details are presented in Figure 1.

### Differences in SARS-CoV-2 Tested and Positive Cohort Characteristics by Preexisting Cognitive Impairment

Based on our criteria (ICD-10 diagnosis codes or medication utilization), we identified 6,364 individuals with preexisting cognitive impairment, of whom 843 (13.2%) tested positive. In addition to being older (mean [SD] age 78.5 [11.2] vs. 50.7 [18.3] years), tested individuals with cognitive impairment (vs. no cognitive impairment) were predominantly male (43.2 vs. 40.3%), either White (71.7 vs. 64.8%) or Black (20.4 vs. 18.4%), separated/widowed/divorced (36.4 vs. 10.4%), Medicare-insured (85.6 vs. 26.5%), residing in more disadvantaged areas (median [IQR] ADI: 41 [21–65] vs. 39 [20–62]), and had a higher overall comorbidity burden (median [IQR] CCI: 8 [6–10] vs. 2 [0–4]; Supplementary Table 1). Among individuals with prior cognitive impairment, a higher cardiovascular (myocardial infarction: 27.5 vs. 7.2%, congestive heart failure: 38.0 vs. 9.7%, hypertension: 89.0 vs. 44.5%), cerebrovascular (stroke or TIA: 44.8 vs. 9.6%), and metabolic (diabetes without complications: 39.7 vs. 19.1%) burden was noted. Similar to the tested cohort, among SARS-CoV-2 positive individuals, significant differences between patients with and without cognitive impairment were also observed for age (mean [SD]: 79.6 [10.9] vs. 48.2 [17.3] years), race (White: 65.0 vs. 61.1% and Black: 25.9 vs. 22.5%), and comorbidity burden (median [IQR] CCI: 7 [6–10] vs. 1 [0–3]) (Supplementary Table 2). Among the SARS-CoV-2 positive cohort, a greater proportion of cognitively impaired (vs. not cognitively impaired) individuals were hospitalized (84.5 vs. 31.5%).

### Factors Associated With SARS-CoV-2 Susceptibility and COVID-19 Mortality

Regardless of a prior history of cognitive impairment, we evaluated factors associated with SARS-CoV-2 susceptibility (Supplementary Table 3) and COVID-19 mortality (Supplementary Table 4). Among tested individuals, male sex (vs. female; OR, 95% CI: 1.18, 1.15–1.22), Black race (vs. White; OR, 95% CI: 1.35, 1.31–1.40), Hispanic ethnicity (vs. Non-Hispanic; OR, 95% CI: 2.54, 2.46–2.62), and higher ADI (OR, 95% CI: 1.01, 1.01–1.01) were related to increased odds of infection. In the COVID-19 hospitalized cohort, higher mortality was seen in patients of advanced age (OR, 95% CI: 1.05, 1.05–1.06), male sex (OR, 95% CI: 1.25, 1.08–1.47), and higher CCI burden (OR, 95% CI: 1.19, 1.17–1.21) (Supplementary Table 4). Increased odds of mortality were also related to critically abnormal vital signs (respiratory rate, temperature, and oxygen saturation), development of particular hospital course complications (pneumonia, acute respiratory distress syndrome, bronchitis, acute renal injury, acute hepatic injury, heart failure, and respiratory failure), abnormal laboratory parameters, and utilization of intensive care and mechanical ventilation resources.

### Propensity Score Matched Analyses

**Figure 2:**
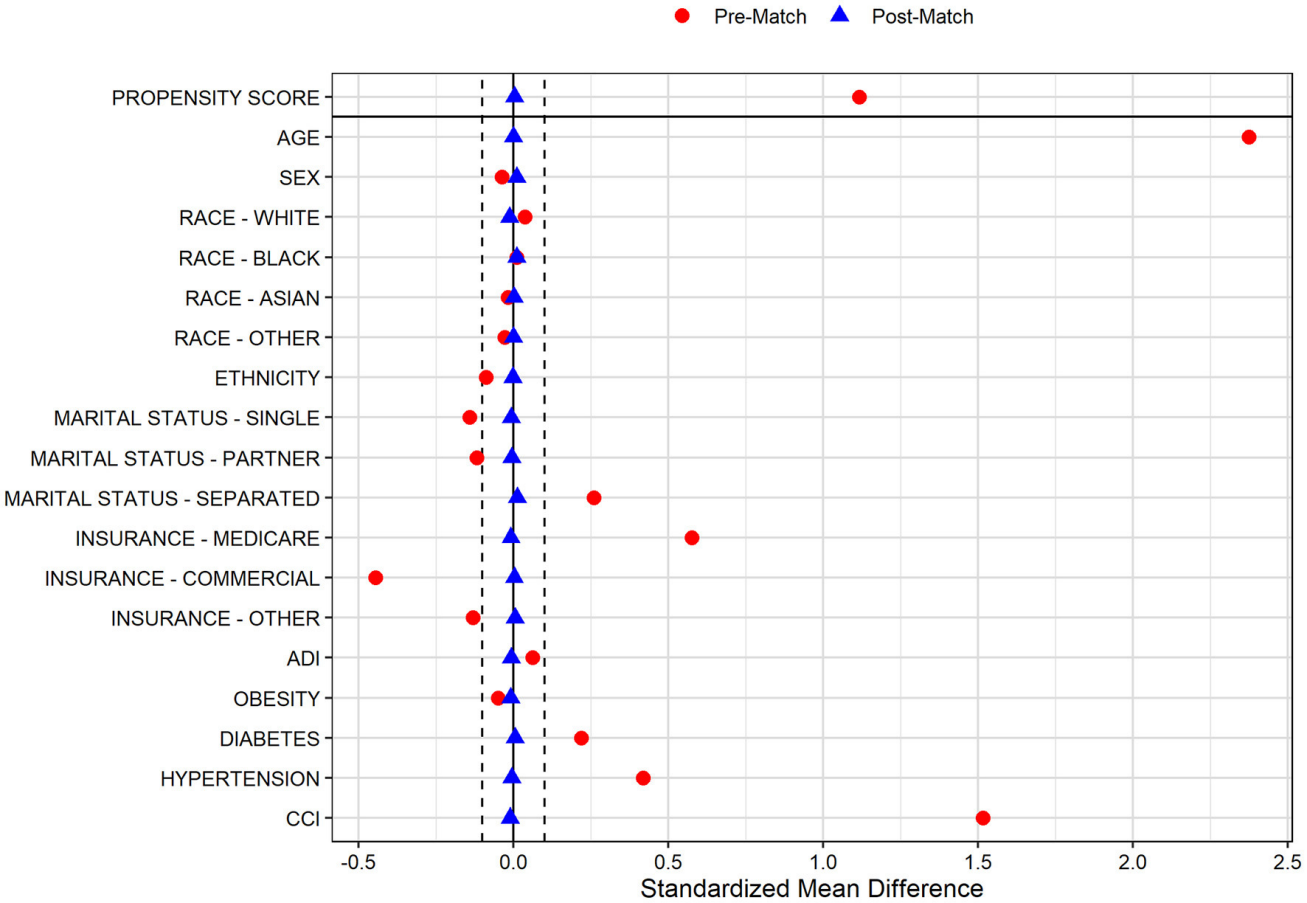
Covariate and propensity score balance for SARS-CoV-2 susceptibility analyses. Pre- and post-matched standardized mean differences (SMD) are presented, using a SMD threshold of 0.1. Cohorts matched on age, sex, race, ethnicity, marital status, insurance coverage, area deprivation index (ADI), obesity, diabetes, hypertension, and overall Charlson Comorbidity Index (CCI).

**Figure 3:**
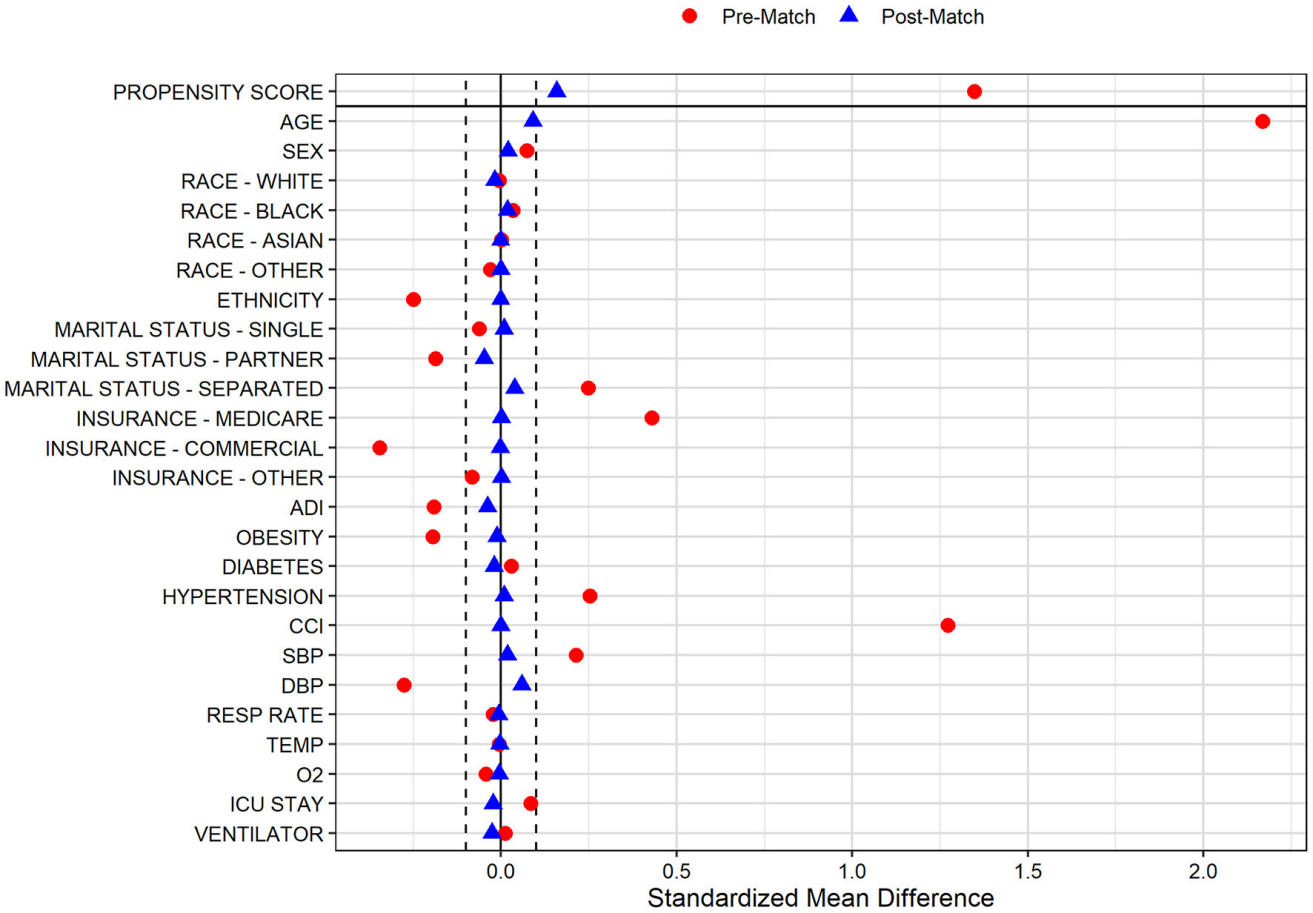
Covariate and propensity score balance for COVID-19 mortality analyses. Pre- and post-matched standardized mean differences (SMD) are presented, using a SMD threshold of 0.1. Cohorts matched on age, sex, race, ethnicity, marital status, insurance coverage, area deprivation index (ADI), obesity, diabetes, hypertension, overall Charlson Comorbidity Index (CCI), mean systolic and diastolic blood pressure (SBP/DBP) at admission, elevated respiratory rate at admission, elevated temperature at admission, low oxygen saturation (O2) at admission, intensive care unit (ICU) stay, and mechanical ventilation utilization.

**Figure 4:**
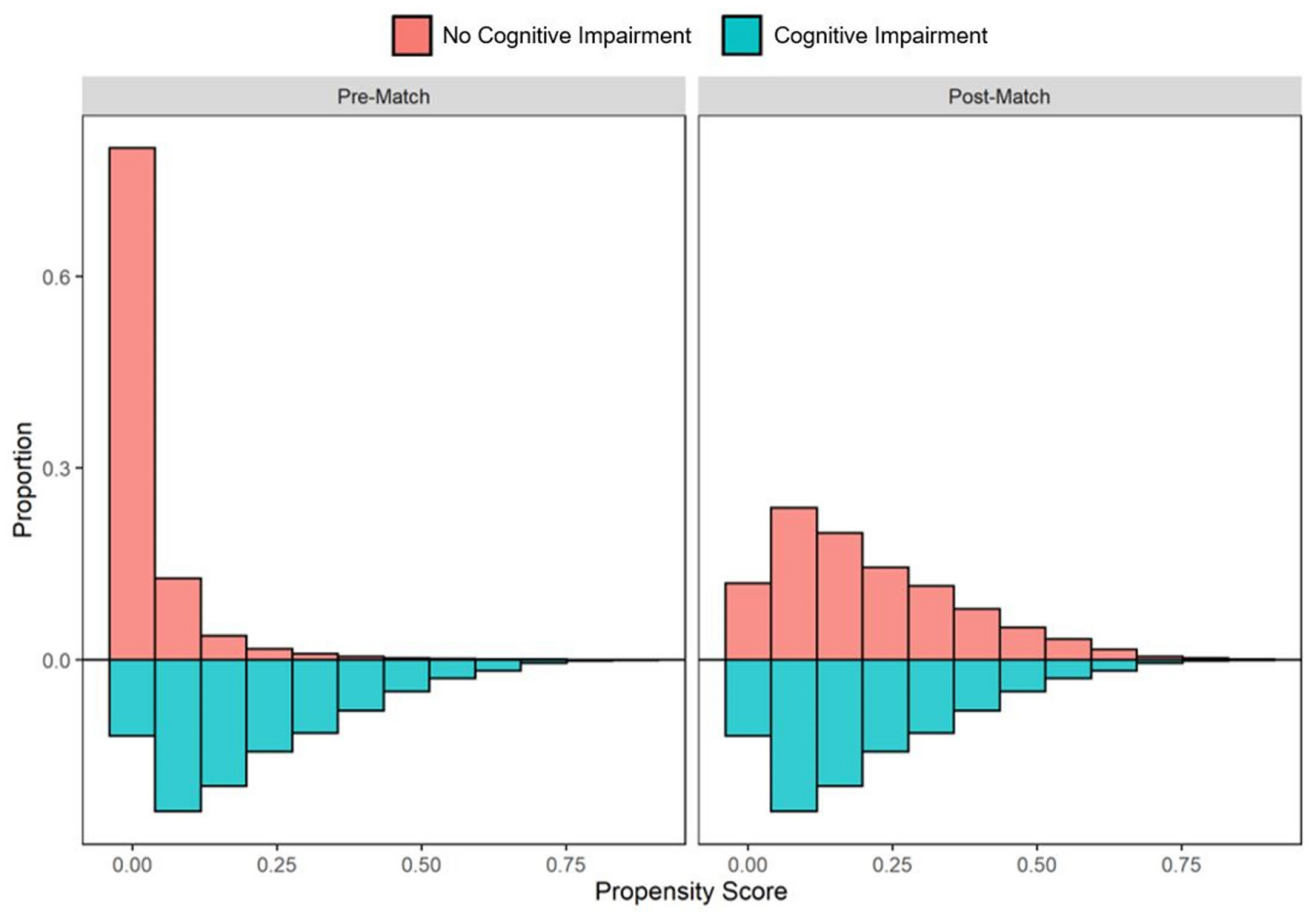
Pre- and post-matched distribution of individual propensity scores among SARS-CoV-2 tested individuals, by preexisting cognitive impairment status. Cohort proportions (*Y*-axis) by propensity score (*X*-axis) are presented. Cohorts matched on age, sex, race, ethnicity, marital status, insurance coverage, area deprivation index (ADI), obesity, diabetes, hypertension, and overall Charlson Comorbidity Index (CCI).

**Figure 5:**
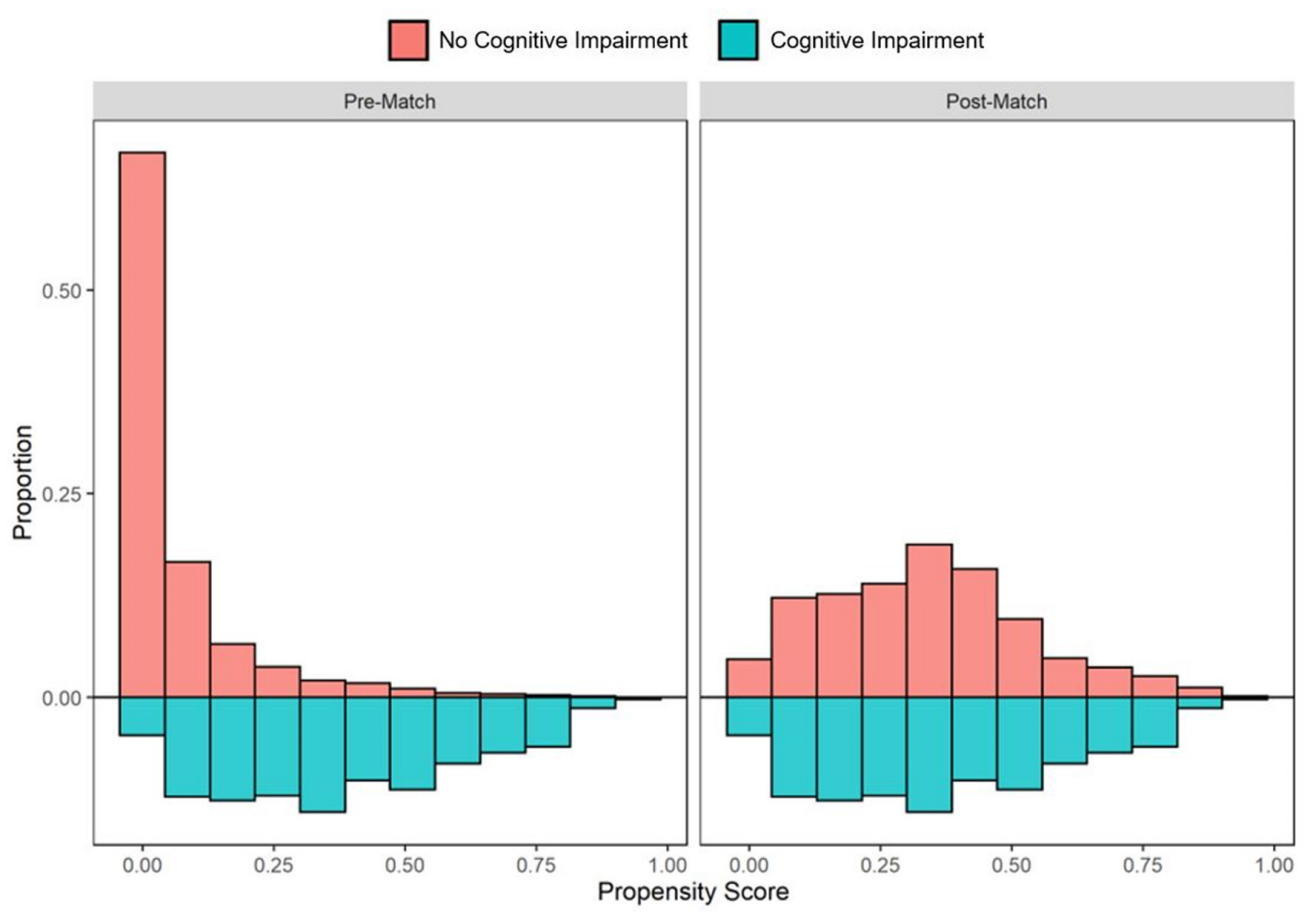
Pre- and post-matched distribution of individual propensity scores among COVID-19 hospitalized patients, by preexisting cognitive impairment status. Cohort proportions (*Y*-axis) by propensity score (*X*-axis) are presented. Cohorts matched on age, sex, race, ethnicity, marital status, insurance coverage, area deprivation index (ADI), obesity, diabetes, hypertension, overall Charlson Comorbidity Index (CCI), mean systolic and diastolic blood pressure (SBP/DBP) at admission, elevated respiratory rate at admission, elevated temperature at admission, low oxygen saturation (O2) at admission, intensive care unit (ICU) stay, and mechanical ventilation utilization.

In the pre-PS matched cohort, cognitive impairment was significantly associated with increased odds of both SARS-CoV-2 susceptibility (OR, 95% CI: 1.12, 1.04–1.21) and COVID-19 mortality (2.54, 2.07–3.12). Adequacy of PS match for both analyses (susceptibility and mortality) was quantitatively assessed by pre- vs. post-matched SMD and visually by evaluating the overlap between pre- and post-matched PS for individuals with and without cognitive impairment. The covariate and PS balance for susceptibility and mortality analyses are illustrated in Figures 2, 3, respectively. The overlap between the pre- and post-matched distribution of individual PS among the SARS-CoV-2 tested and COVID-19 hospitalized cohorts are presented in Figures 4, 5, respectively.

### Propensity Score Analyses for SARS-CoV-2 Susceptibility

Among all tested individuals with prior cognitive impairment, 6,192 of 6,364 (97.3%) were one-to-one matched with individuals without cognitive impairment on PS model-included socio-demographic and comorbidity characteristics. The pre- and post-matched comparison demonstrates that covariate balance was adequately achieved for all included factors with significant SMD reduction in the post-matched sample (Figure 2). In particular, pre-match differences were balanced for age (mean [SD]: 78.4 [11.2] vs. 78.4 [10.7] years), marital status (single: 15.8 vs. 16.5%; married: 47.1 vs. 47.7%; separated: 37.1 vs. 35.9%), Medicare coverage (85.6 vs. 86.4%), CCI burden (median [IQR]: 8 [6–11] vs. 8 [5–11]), diabetes (42.7 vs. 42.2%), and hypertension (89.1 vs. 89.7%; Table 1). Whereas the pre-match SARS-CoV-2 infection rate in individuals with and without cognitive impairment was 13.1 and 12.0%, respectively, the infection rate for the cohort without cognitive impairment after matching was 9.1%. The post-matched likelihood of SARS-CoV-2 susceptibility among those with cognitive impairment (vs. without cognitive impairment) was observed to strengthen, with a 51% higher likelihood of SARS-CoV-2 infection among individuals with cognitive impairment (OR, 95% CI: 1.51, 1.35–1.70).

**Table 1:** Pre- and post-propensity score matched characteristics of adults tested for SARS-CoV-2 at Houston Methodist through December 11th, 2020, by cognitive impairment history.

	**No cognitive impairment**		**Cognitively impaired**	***P*-value^**b**^**
	**Pre-match (*n* = 173,615)**	**Post-match (*n* = 6,192)**	**Post-match (*n* = 6,192)^**a**^**	
**Demographic and social characteristics**				
Age—mean (SD)	50.7 (18.3)	78.4 (10.7)	78.4 (11.2)	0.997
Female (vs. Male)—*n* (%)	103,716 (59.7)	3,451 (55.7)	3,512 (56.7)	0.277
**Race—*****n*** **(%)**				
White	112,416 (64.8)	4,550 (73.5)	4,470 (72.2)	0.111
Black	31,880 (18.4)	1,201 (19.4)	1,271 (20.5)	0.121
Asian	10,522 (6.1)	272 (4.4)	280 (4.5)	0.761
Other	18,797 (10.8)	169 (2.7)	171 (2.8)	0.956
Hispanic (vs. Non-Hispanic) – *n* (%)	32,495 (18.7)	690 (11.1)	675 (10.9)	0.688
**Marital status**^**c**^**–*****n*** **(%)**				
Single	49,091/163,139 (30.1)	1,020 (16.5)	976 (15.8)	0.293
Married/life partner/common law	96,071/163,139 (58.9)	2,952 (47.7)	2,916 (47.1)	0.529
Separated/divorced/widowed	17,977/163,139 (11.0)	2,220 (35.9)	2,300 (37.1)	0.140
**Insurance type—*****n*** **(%)**				
Medicare	45,936 (26.5)	5,352 (86.4)	5,302 (85.6)	0.204
Commercial	84,531 (48.7)	363 (5.9)	379 (6.1)	0.570
Other	43,148 (24.9)	477 (7.7)	511 (8.3)	0.274
Area Deprivation Index—median (IQR)	39 (20–62)	41 (22–66)	41 (21–65)	0.663
**Comorbidities and coexisting conditions**				
Charlson Comorbidity Index (CCI) score—median (IQR)	2 (0–4)	8 (5–11)	8 (6–11)	0.556
Obesity—*n* (%)	51,512 (29.7)	1,702 (27.5)	1,651 (26.7)	0.312
Diabetes—*n* (%)	34,177 (19.7)	2,615 (42.2)	2,647 (42.7)	0.573
Hypertension—*n* (%)	77,275 (44.5)	5,554 (89.7)	5,518 (89.1)	0.307
**SARS-CoV-2 positive—*****n*** **(%)**	20,764 (12.0)	561 (9.1)	811 (13.1)	<0.001
	**Pre-match**		**Post-match**	
**Susceptibility OR (95% CI)**^d^	**1.12 (1.04–1.21)**		**1.51 (1.35–1.70)**	

a *Excludes 172 (2.7%) tested individuals with preexisting cognitive impairment due to missing-ness of data to match on*.

b *Mean, proportional, and median difference between post-match cohorts*.

c *Marital status unavailable for 10,476 (6.0%) tested individuals in pre-match cohort*.

d *SARS-CoV-2 susceptibility odds ratio (95% confidence interval) for tested individuals with preexisting cognitive impairment*.

### Propensity Score Analyses for COVID-19 Mortality

In the COVID-19 hospitalization cohort, 687 of 712 (96.5%) patients with cognitive impairment were one-to-one PS matched across demographic and clinical variables with individuals without cognitive impairment. Similar to the tested cohort, adequate balance across all prehospital and hospitalization covariates was achieved (Figure 3). The PS balanced covariates for include age (mean [SD]: 80.1 [10.6] vs. 79.2 [9.6] years), females (55.5 vs. 53.4%) marital status (single: 21.0 vs. 20.1%; married: 39.9 vs. 44.7%; separated: 39.2 vs. 35.2%), Medicare coverage (77.9 vs. 77.7%), CCI burden (median [IQR]: 8 [6–10] vs. 8 [5–11]), admission to intensive care (38.6 vs. 40.9%), and use of mechanical ventilation (17.5 vs. 20.1%; Table 2). Prior to matching, the mortality rate in the hospitalized cohort of patients without cognitive impairment was 8.7%; however, the mortality rate in the matched sample was 20.1% (compared with 19.4% among patients with cognitive impairment; Table 2). The post-matched differences in mortality outcomes were not observed to be significant for cognitively impaired individuals (vs. no cognitive impairment), OR (95% CI): 0.96 (0.73–1.25).

**Table 2:** Pre- and post-propensity score matched characteristics of adults hospitalized with COVID-19 at Houston Methodist through December 11th, 2020, by cognitive impairment history.

	**No cognitive impairment**		**Cognitively impaired**	
	**Pre-match (*n* = 6,536)**	**Post-match (*n* = 687)**	**Post-match (*n* = 687)^**a**^**	***P*-value^b^**
**Demographic and social characteristics**				
Age—mean (SD)	57.2 (16.5)	79.2 (9.6)	80.1 (10.6)	0.079
Female (vs. Male)—*n* (%)	3,148 (48.2)	367 (53.4)	381 (55.5)	0.481
**Race—*****n*** **(%)**				
White	4,262 (65.2)	462 (67.2)	450 (65.5)	0.530
Black	1,436 (22.0)	163 (23.7)	176 (25.6)	0.453
Asian	319 (4.9)	36 (5.2)	35 (5.1)	1.000
Other	519 (7.9)	26 (3.8)	26 (3.8)	1.000
Hispanic (vs. Non-Hispanic)—*n* (%)	2,576 (39.4)	104 (15.1)	103 (15.0)	1.000
**Marital status**^**c**^**–*****n*** **(%)**				
Single	1,735/6,362 (27.3)	138 (20.1)	144 (21.0)	0.738
Married/life partner/common law	3,720/6,362 (58.5)	307 (44.7)	274 (39.9)	0.081
Separated/divorced/widowed	907/6,362 (14.3)	242 (35.2)	269 (39.2)	0.147
**Insurance type—*****n*** **(%)**				
Medicare	2,275 (34.8)	534 (77.7)	535 (77.9)	1.000
Commercial	2,464 (37.7)	24 (3.5)	22 (3.2)	0.881
Other	1,797 (27.5)	129 (18.8)	130 (18.9)	1.000
Area Deprivation Index—median (IQR)	54 (33–74)	47 (26–73)	45 (24–71)	0.480
**Comorbidities and coexisting conditions**				
Charlson Comorbidity Index (CCI) score—median (IQR)	3 (1–6)	8 (5–11)	8 (6–10)	0.994
Obesity—*n* (%)	2,676 (40.9)	158 (23.0)	150 (21.8)	0.651
Diabetes—*n* (%)	2,841 (43.5)	334 (48.6)	321 (46.7)	0.517
Hypertension—*n* (%)	4,311 (66.0)	623 (90.7)	629 (91.6)	0.635
**Vital signs at hospital admission**				
SBP (mmHg)—mean (SD)	130.7 (17.7)	134.7 (19.1)	135.0 (19.8)	0.736
DBP (mmHg)—mean (SD)	71.8 (9.2)	68.9 (8.4)	69.4 (8.7)	0.265
Respiratory rate ≥ 24 breath/min—*n* (%)	1,107 (16.9)	105 (15.3)	100 (14.6)	0.762
Temperature ≥ 38°C—*n* (%)	144 (2.2)	15 (2.2)	12 (1.7)	0.697
Oxygen saturation <94%—*n* (%)	1,237 (19.0)	104 (15.1)	100 (14.6)	0.762
**Hospital acuity of care**				
ICU admission—*n* (%)	1,969 (30.1)	281 (40.9)	265 (38.6)	0.408
Invasive mechanical ventilation—*n* (%)	1,064 (16.3)	138 (20.1)	120 (17.5)	0.240
**In-hospital mortality**—***n*** **(%)**	569 (8.7)	138 (20.1)	133 (19.4)	0.786
	**Pre-match**		**Post-match**	
**In-hospital mortality OR (95% CI)**^**d**^	**2.54 (2.07**–**3.12)**		**0.96 (0.73**–**1.25)**	

a *Excludes 25 (3.5%) COVID-19 hospitalized patients with preexisting cognitive impairment due to missing-ness of data to match on*.

b *Mean, proportional, and median difference between post-match cohorts*.

c *Marital status unavailable for 174 (2.7%) tested individuals in pre-match cohort*.

d *In-hospital mortality odds ratio (95% confidence interval) for COVID-19 patients with preexisting cognitive impairment*.

*SBP, Systolic Blood Pressure; DBP, Diastolic Blood Pressure; ICU, Intensive Care Unit*.

### Sex-Stratified Propensity Score Analyses for SARS-CoV-2 Susceptibility and COVID-19 Mortality

Sex-stratified analyses were also performed to evaluate conditional independence between prior cognitive impairment and study outcomes (SARS-CoV-2 infection and in-hospital COVID-19 mortality). SARS-CoV-2 susceptibility was higher for males (OR, 95% CI: 1.53, 1.29–1.82), compared to females (OR, 95% CI: 1.50, 1.28–1.74) (Cochran–Mantel–Haenszel *P* < 0.001). In-hospital mortality was not demonstrated to differ across sex strata (Cochran–Mantel–Haenszel *P* = 0.393).

## Discussion

Our data demonstrate a strong and independent association between higher SARS-CoV-2 infection susceptibility and preexisting cognitive impairment in a carefully balanced and matched cohort across several important sociodemographic and comorbidity covariates. This study leveraged a robust patient registry (CURATOR) (11) and is especially significant for the inclusion of a broad range of clinical and community level factors. In the matched sample, cognitively impaired individuals were 51% more likely to contract SARS-CoV-2 infection compared to those without a prior history of cognitive impairment. However, similar significant differences in higher risk of COVID-19 associated mortality were not observed among cognitively impaired individuals in the fully matched sample. Yet, individuals with cognitive impairment had a higher mortality likelihood in the unadjusted analyses. We see that overall, cognitively impaired individuals were older and had a higher cardiovascular and cerebrovascular comorbidity burden. These findings are in line with reporting that upon hospitalization, older patients with coexisting conditions and requiring critical care are among the sub-population most at risk of poor outcomes (14, 15).

The observed, increased risk of SARS-CoV-2 infection among the population with cognitive impairment may, in part, be attributed to a general lack of cognitive awareness or physical capacity to adhere to public health guidance about prevention measures (9). Prior studies have highlighted experiences of older adults with cognitive impairment during the COVID-19 pandemic with an emphasis on the high prevalence of sense of distress and precarity as they attempted to adapt to pandemic-related restrictions (e.g., sheltering-in-place) (16, 17). Furthermore, patients relying on managed care or social support may have difficulty adapting to social distancing requirements or visitation restrictions. On the other hand, for individuals with cognitive impairment that are actively being cared for, the risks are compounded because caregivers may constitute a potential source of infection. Caregivers who live in likely require leaving the home to meet the basic needs of the individual; those who do not live within the home risk blending safety “bubbles.” Of importance to note, we were unable to collect information related to nursing home residence, long-term care, or other institutionalization; therefore, our analyses do not account for the potential of congregate settings as an additional exposure contributing to increased SARS-CoV-2 infection (18–20).

For the cognitively impaired population, isolation in limited or unfamiliar settings can also lead to increased risk for onset of acute conditions, such as delirium, as well as poor outcomes (9). To our knowledge, there are no prior reports of association between cognitive impairment and in-hospital mortality; however, an analysis from a study of 30-day all-cause mortality among patients relying on post-acute or long-term care demonstrated a >2-fold increased odds of death for residents with cognitive impairment (21). Higher SARS-CoV-2 susceptibility and COVID-19 mortality is reported among older adults, which may confound the independent relationship between cognitive impairment and likelihood of SARS-CoV-2 infection and poor outcomes; however, we attempted to account for this through carefully matched analyses. Given the results of our analyses, it is possible that in our cohort of hospitalized COVID-19 patients, preexisting cognitive impairment did not confer additional risk of mortality beyond that attributable to increased age and higher overall comorbidity burden.

An estimated one-third of older adults impacted with mild cognitive impairment, dementia or Alzheimer's disease live alone in the United States (22). Prior research has suggested that this sub-population may be at further risk of consequence, ranging from both a lack of awareness of the diagnosis to a lack of preparation to manage one's health on their own (22, 23). Understanding this risk is particularly relevant to the cognitively impaired population we identified, in which >50% had a self-reported marital status of single, separated, divorced, or widowed. It has been widely recommended that individuals diagnosed with cognitive impairment receive tailored care, and this guidance is particularly important during public health crises—such as pandemics or extreme weather disasters—when access to home care providers or aides may be limited (16). The COVID-19 pandemic brought about a substantial rise in utilization of telehealth services (24), and this shift in care delivery presented an additionally unique challenge and opportunity for the care of the cognitively impaired population (25, 26). The lessons learned throughout the pandemic underscore the crucial need to develop strategies that ensure safe and continued support of this vulnerable population during similar public health crises.

Limitations of our study include analysis of data from a single healthcare system across the greater Houston metropolitan area as well as potential misclassification of cognitive impairment (false negatives). Our cohort definition did not allow for a staging of the severity of cognitive impairments, although the mean age in our matched propensity analyses suggested an advanced age with higher likelihood of dementia. Though we analyzed a large diverse sample, it may not be nationally representative, and management practices across different healthcare systems can influence both infection screening and mortality metrics. Reliance on diagnosis codes to extract medical history and comorbidity information may not comprehensively capture all prior evidence of cognitive impairment; however, we attempted to broaden our search by including administration of cognitive impairment-specific medications, which has been reported to significantly increase the validity of identifying patients with prior cognitive impairment (12). Nonetheless, the possibility of undiagnosed or misclassified cognitive impairment still exists. Moreover, this study focused on evaluating the clinical and socio-demographic factors that predispose individuals with preexisting cognitive impairment to SARS-CoV-2 infection; however, behavioral factors and current functional capacity likely play an important role in susceptibility among older and cognitively impaired individuals. We did not have data on prior living situation of the study participants or their receipt of formal or informal caregiving; however, we did evaluate other pertinent socio-economic variables, and included community-level factors often neglected in clinical research. Expanded research will be necessary to precisely understand how socio-behavioral factors influence the modes of disease transmission among this vulnerable population as well as how neuro-biological and anatomical attributes of the nervous system among the cognitively impaired independently or additively increase SARS-CoV-2 susceptibility. With respect to our study outcomes, differential behaviors by individuals or their providers may influence the frequency of testing. Lastly, our evaluation of COVID-19 outcomes also focused on in-hospital mortality; follow-up studies are needed to assess the long-term effects of SARS-CoV-2 infection and prolonged hospitalization and post-hospitalization care, which may have disproportionately higher burden of poor neurological and functional outcomes among those with preexisting cognitive impairment.

Notwithstanding these limitations, our findings underscore the importance of evaluating specific social, behavioral, and biological pathways leading to higher SARS-CoV-2 susceptibility among individuals with cognitive impairment. Furthermore, we highlight the utility of patient registries, such as CURATOR, to identify risk groups. The disparate burden of COVID-19 and similar future public health crises among the cognitively impaired population is likely to be high. Strategies for early identification and targeted prevention—such as through regular screening, education and awareness, and behavioral reinforcement of public health practices (e.g., washing hands, wearing masks, and social distancing) to mitigate disease transmission—are necessary. Risk-stratified comorbidity management is also warranted for this high-risk and vulnerable population.

## Data Availability Statement

The datasets presented in this article are not readily available because data was collected under the Houston Methodist IRB-approved CURATOR project. Reasonable requests for a fully de-identified dataset can be made to the corresponding author. These requests will be evaluated on an individual basis by the CURATOR governance committee and the institutional review board. Requests to access the datasets should be directed to Farhaan Vahidy (fvahidy@houstonmethodist.org).

## Ethics Statement

The studies involving human participants were reviewed and approved by Houston Methodist Institutional Review Board. Written informed consent for participation was not required for this study in accordance with the national legislation and the institutional requirements.

## Author Contributions

FV was responsible for the study conception and design. Data acquisition and analysis were performed by AP. Interpretation of results and initial drafting of the manuscript were completed by AP and FV. All authors contributed to critical revision of the manuscript and approval of the final version for submission.

## Conflict of Interest

The authors declare that the research was conducted in the absence of any commercial or financial relationships that could be construed as a potential conflict of interest.
