# Supplementary material for: SARS-CoV-2 Susceptibility and COVID-19 Mortality Among Older Adults With Cognitive Impairment: Cross-Sectional Analysis From Hospital Records in a Diverse US Metropolitan Area
Source: Front Neurol. 2021 Jul 22;12:692662. doi: 10.3389/fneur.2021.692662 (PMC8344862; doi:10.3389/fneur.2021.692662)
Supplement: Supplementary file 1 [file Table_1.docx]

Supplementary Material

**Supplementary Table 1.** Socio-demographic and comorbidity characteristics of adults tested for SARS-CoV-2 at Houston Methodist through December 11^th^, 2020, by cognitive impairment history

|  | **Cognitively Impaired**  *(n = 6,364)* | **No Cognitive Impairment**  *(n = 173,615)* | ***P-value*** |
| --- | --- | --- | --- |
| **Demographic and Social Characteristics – n (%)** | | | |
| Age – mean (SD) | 78.5 (11.2) | 50.7 (18.3) | <0.001 |
| Age Categories |  |  |  |
| 18-35 | 33 (0.5) | 44,445 (25.6) | <0.001 |
| 36-50 | 119 (1.9) | 40,842 (23.5) | <0.001 |
| 51-65 | 521 (8.2) | 44,987 (25.9) | <0.001 |
| 66-80 | 2,712 (42.6) | 36,013 (20.7) | <0.001 |
| >80 | 2,979 (46.8) | 7,328 (4.2) | <0.001 |
| Female (vs. Male) | 3,616 (56.8) | 103,716 (59.7) | <0.001 |
| Race |  |  |  |
| White | 4,566 (71.7) | 112,416 (64.8) | <0.001 |
| Black | 1,300 (20.4) | 31,880 (18.4) | <0.001 |
| Asian | 291 (4.6) | 10,522 (6.1) | <0.001 |
| Other / Mixed / Not Reported | 207 (3.3) | 18,797 (10.8) | <0.001 |
| Hispanic (vs. Non-Hispanic) | 691 (10.9) | 32,495 (18.7) | <0.001 |
| Marital Status |  |  |  |
| Single | 989 (15.5) | 49,091 (28.3) | <0.001 |
| Married / Life Partner / Common Law | 2,957 (46.5) | 96,071 (55.3) | <0.001 |
| Separated / Divorced / Widowed | 2,319 (36.4) | 17,977 (10.4) | <0.001 |
| Unavailable | 99 (1.6) | 10,476 (6.0) | <0.001 |
| Insurance Type |  |  |  |
| Commercial | 386 (6.1) | 84,531 (48.7) | <0.001 |
| Medicare | 5,450 (85.6) | 45,936 (26.5) | <0.001 |
| Medicaid | 80 (1.3) | 6,978 (4.0) | <0.001 |
| Self-Pay | 57 (0.9) | 31,698 (18.3) | <0.001 |
| Other | 391 (6.1) | 4,472 (2.6) | <0.001 |
| Area Deprivation Index (ADI) – median (IQR) | 41 (21 – 65) | 39 (20 – 62) | <0.001 |
| Median Household Income – median (IQR) | 68,318 (53,116 – 99,276) | 70,658 (53,633 – 100,217) | <0.001 |
| Percent Living in Poverty – median (IQR) | 10.4 (6.0 – 17.1) | 10.3 (6.0 – 16.3) | 0.137 |
| Population Density (pop/mi^2^) – median (IQR) | 2,797 (1,429 – 4,261) | 2,797 (1,408 – 4,265) | 0.432 |
| **Comorbidities and Co-existing Conditions / Prior Vaccinations – n (%)** | | | |
| Charlson Comorbidity Index (CCI) Score – median (IQR) | 8 (6 – 10) | 2 (0 – 4) | <0.001 |
|  |  |  |  |
| Myocardial Infarction | 1,749 (27.5) | 12,558 (7.2) | <0.001 |
| Congestive Heart Failure | 2,417 (38.0) | 16,857 (9.7) | <0.001 |
| Peripheral Vascular Disease | 1,996 (31.4) | 18,031 (10.4) | <0.001 |
| CVA / TIA | 2,851 (44.8) | 16,712 (9.6) | <0.001 |
| COPD | 2,099 (33.0) | 33,588 (19.3) | <0.001 |
| Connective Tissue Disease | 389 (6.1) | 6,087 (3.5) | <0.001 |
| Peptic Ulcer | 463 (7.3) | 5,332 (3.1) | <0.001 |
| Liver Disease (Mild) | 645 (10.1) | 14,916 (8.6) | <0.001 |
| Liver Disease (Moderate to Severe) | 142 (2.2) | 2,200 (1.3) | <0.001 |
| Diabetes w/o Complications | 2,529 (39.7) | 33,195 (19.1) | <0.001 |
| Diabetes with Complications | 1,529 (24.0) | 11,508 (6.6) | <0.001 |
| Hemiplegia | 517 (8.1) | 2,629 (1.5) | <0.001 |
| CKD (Mild to Moderate) | 729 (11.5) | 6,248 (3.6) | <0.001 |
| Solid Tumor (Localized) | 1,263 (19.8) | 16,601 (9.6) | <0.001 |
| Solid Tumor (Metastatic) | 930 (14.6) | 12,940 (7.5) | <0.001 |
| AIDS | 40 (0.6) | 1,058 (0.6) | 0.912 |
|  |  |  |  |
| BMI – mean (SD) | 26.0 (6.2) | 29.8 (7.3) | <0.001 |
| Obesity | 1,677 (26.4) | 51,512 (29.7) | <0.001 |
| Hypertension | 5,665 (89.0) | 77,275 (44.5) | <0.001 |
| Hyperlipidemia | 4,555 (71.6) | 61,469 (35.4) | <0.001 |
| Asthma | 716 (11.3) | 19,573 (11.3) | 0.971 |
| Smoking (Current/Former) * | 2,421/6,011 (40.3) | 45,667/147,178 (31.0) | <0.001 |
| Prior Vaccination * |  |  |  |
| Influenza | 3,662/4,917 (74.5) | 44,138/74,351 (59.4) | <0.001 |
| Pneumonia | 3,930/4,874 (80.6) | 34,242/80,812 (42.4) | <0.001 |
|  |  |  |  |
| SARS-CoV-2 Positive | 843 (13.2) | 20,764 (12.0) | 0.002 |
| ** Denominator denotes number of non-missing values.* | | | |

**Supplementary Table 2.** Socio-demographic and comorbidity characteristics of adults testing positive for SARS-CoV-2 at Houston Methodist through December 11^th^, 2020, by cognitive impairment history

|  | **Cognitively Impaired**  *(n = 843)* | **No Cognitive Impairment**  *(n = 20,764)* | ***P-value*** |
| --- | --- | --- | --- |
| **Demographic and Social Characteristics – n (%)** | | | |
| Age – mean (SD) | 79.6 (10.9) | 48.2 (17.3) | <0.001 |
| Age Categories |  |  |  |
| 18-35 | 4 (0.5) | 5,797 (27.9) | <0.001 |
| 36-50 | 10 (1.2) | 5,696 (27.4) | <0.001 |
| 51-65 | 57 (6.8) | 5,427 (26.1) | <0.001 |
| 66-80 | 342 (40.6) | 3,230 (15.6) | <0.001 |
| >80 | 430 (51.0) | 614 (3.0) | <0.001 |
| Female (vs. Male) | 474 (56.2) | 11,637 (56.0) | 0.944 |
| Race |  |  |  |
| White | 548 (65.0) | 12,690 (61.1) | 0.025 |
| Black | 218 (25.9) | 4,670 (22.5) | 0.024 |
| Asian | 38 (4.5) | 1,141 (5.5) | 0.246 |
| Other / Mixed / Not Reported | 39 (4.6) | 2,263 (10.9) | <0.001 |
| Hispanic (vs. Non-Hispanic) | 129 (15.3) | 7,145 (34.4) | <0.001 |
| Marital Status |  |  |  |
| Single | 170 (20.2) | 7,135 (34.4) | <0.001 |
| Married / Life Partner / Common Law | 334 (39.6) | 10,899 (52.5) | <0.001 |
| Separated / Divorced / Widowed | 314 (37.2) | 1,833 (8.8) | <0.001 |
| Unavailable | 25 (3.0) | 897 (4.3) | 0.069 |
| Insurance Type |  |  |  |
| Commercial | 33 (3.9) | 10,529 (50.7) | <0.001 |
| Medicare | 666 (79.0) | 4,018 (19.4) | <0.001 |
| Medicaid | 15 (1.8) | 967 (4.7) | <0.001 |
| Self-Pay | 6 (0.7) | 4,765 (22.9) | <0.001 |
| Other | 123 (14.6) | 485 (2.3) | <0.001 |
| Area Deprivation Index (ADI) – median (IQR) | 45 (24 – 70) | 48 (27 – 70) | 0.068 |
| Median Household Income – median (IQR) | 68,318 (50,465 – 94,494) | 66,450 (50,035 – 92,166) | 0.081 |
| Percent Living in Poverty – median (IQR) | 11.3 (6.3 – 17.5) | 12.0 (6.9 – 17.8) | 0.024 |
| Population Density (pop/mi^2^) – median (IQR) | 2,814 (1,439 – 4,352) | 2,886 (1,439 – 4,300) | 0.410 |
| **Comorbidities and Co-existing Conditions / Prior Vaccinations – n (%)** | | | |
| Charlson Comorbidity Index (CCI) Score – median (IQR) | 7 (6 – 10) | 1 (0 – 3) | <0.001 |
|  |  |  |  |
| Myocardial Infarction | 237 (28.1) | 1,355 (6.5) | <0.001 |
| Congestive Heart Failure | 320 (38.0) | 1,642 (7.9) | <0.001 |
| Peripheral Vascular Disease | 243 (28.8) | 1,435 (6.9) | <0.001 |
| CVA / TIA | 338 (40.1) | 1,407 (6.8) | <0.001 |
| COPD | 257 (30.5) | 3,584 (17.3) | <0.001 |
| Connective Tissue Disease | 41 (4.9) | 509 (2.5) | <0.001 |
| Peptic Ulcer | 45 (5.3) | 409 (2.0) | <0.001 |
| Liver Disease (Mild) | 70 (8.3) | 1,468 (7.1) | 0.195 |
| Liver Disease (Moderate to Severe) | 14 (1.7) | 200 (1.0) | 0.068 |
| Diabetes w/o Complications | 366 (43.4) | 4,679 (22.5) | <0.001 |
| Diabetes with Complications | 228 (27.0) | 1,418 (6.8) | <0.001 |
| Hemiplegia | 68 (8.1) | 205 (1.0) | <0.001 |
| CKD (Mild to Moderate) | 116 (13.8) | 756 (3.6) | <0.001 |
| Solid Tumor (Localized) | 136 (16.1) | 1,138 (5.5) | <0.001 |
| Solid Tumor (Metastatic) | 94 (11.2) | 870 (4.2) | <0.001 |
| AIDS | 3 (0.4) | 133 (0.6) | 0.422 |
|  |  |  |  |
| BMI – mean (SD) | 25.7 (6.2) | 31.2 (7.6) | <0.001 |
| Obesity | 189 (22.4) | 5,416 (26.1) | 0.019 |
| Hypertension | 759 (90.0) | 8,558 (41.2) | <0.001 |
| Hyperlipidemia | 553 (65.6) | 6,138 (29.6) | <0.001 |
| Asthma | 77 (9.1) | 2,108 (10.2) | 0.367 |
| Smoking (Current/Former) * | 272/760 (35.8) | 3,987/17,502 (22.8) | <0.001 |
| Prior Vaccination * |  |  |  |
| Influenza | 424/579 (73.2) | 4,322/7,616 (56.7) | <0.001 |
| Pneumonia | 464/589 (78.8) | 3,167/8,572 (36.9) | <0.001 |
|  |  |  |  |
| Hospitalized | 712 (84.5) | 6,536 (31.5) | <0.001 |
| In-Hospital Mortality | 139 (19.5) | 569 (8.7) | <0.001 |
| ** Denominator denotes number of non-missing values.* | | | |

**Supplementary Table 3.** Baseline socio-demographic and comorbidity factors associated with SARS-CoV-2 infection among adults tested through December 11^th^, 2020

|  | **SARS-CoV-2 Positive**  *(n = 21,607)* | **SARS-CoV-2 Negative**  *(n = 158,372)* | **OR (95% CI) ^1^** |
| --- | --- | --- | --- |
| **Demographic and Social Characteristics – n (%)** | | | |
| Age – mean (SD) | 49.4 (18.2) | 52.0 (18.9) | 0.99 (0.99 – 9.99)  P<0.001 |
| Female (vs. Male) | 12,111 (56.1) | 95,221 (60.1) | 0.85 (0.82 – 0.87)  P<0.001 |
| Race |  |  |  |
| White | 13,238 (61.3) | 103,744 (65.5) | *Reference* |
| Black | 4,888 (22.6) | 28,292 (17.9) | 1.35 (1.31 – 1.40)  P<0.001 |
| Asian | 1,179 (5.5) | 9,634 (6.1) | 0.96 (0.90 – 1.02)  P=0.194 |
| Other / Mixed / Not Reported | 2,302 (10.7) | 16,702 (10.5) | 1.08 (1.03 – 1.13)  P=0.001 |
| Hispanic (vs. Non-Hispanic) | 7,274 (33.7) | 25,912 (16.4) | 2.54 (2.46 – 2.62)  P<0.001 |
| Marital Status |  |  |  |
| Single | 7,305 (33.8) | 42,775 (27.0) | *Reference* |
| Married / Life Partner / Common Law | 11,233 (52.0) | 87,795 (55.4) | 0.75 (0.73 – 0.77)  P<0.001 |
| Separated / Divorced / Widowed | 2,147 (9.9) | 18,149 (11.5) | 0.69 (0.66 – 0.73)  P<0.001 |
| Unavailable | 922 (4.3) | 9,653 (6.1) | 0.56 (0.52 – 0.60)  P<0.001 |
| Insurance Type |  |  |  |
| Commercial | 10,562 (48.9) | 74,355 (46.9) | *Reference* |
| Medicare | 4,684 (21.7) | 46,702 (29.5) | 0.71 (0.68 – 0.73)  P<0.001 |
| Medicaid | 982 (4.5) | 6,076 (3.8) | 1.14 (1.06 – 1.22)  P<0.001 |
| Self-Pay | 4,771 (22.1) | 26,984 (17.0) | 1.24 (1.20 – 1.29)  P<0.001 |
| Other | 608 (2.8) | 4,255 (2.7) | 1.01 (0.92 – 1.10)  P=0.894 |
| Area Deprivation Index (ADI) | 49.0 (26.7) | 41.5 (26.6) | 1.01 (1.01 – 1.01)  P<0.001 |
| Zip Code Residence: Low Income *^2^* | 10,433 (48.7) | 61,789 (39.5) | 1.45 (1.41 – 1.50)  P<0.001 |
| Zip Code Residence: High Poverty *^3^* | 10,212 (47.6) | 59,739 (38.1) | 1.47 (1.43 – 1.52)  P<0.001 |
| Zip Code Residence: High Pop Density *^4^* | 9,368 (43.7) | 62,040 (39.6) | 1.18 (1.15 – 1.22)  P<0.001 |
|  | | | |
| Charlson Comorbidity Index (CCI) Score | 2.5 (3.3) | 3.1 (3.6) | 0.95 (0.95 – 0.95)  P<0.001 |
| BMI | 30.9 (7.6) | 29.5 (7.3) | 1.02 (1.02 – 1.03)  P<0.001 |
| Preexisting Cognitive Impairment | 843 (3.9) | 5,521 (3.5) | 1.12 (1.04 – 1.21)  P=0.002 |
| Obesity | 5,605 (25.9) | 47,584 (30.0) | 0.82 (0.79 – 0.84)  P<0.001 |
| Hypertension | 9,317 (43.1) | 73,623 (46.5) | 0.87 (0.85 – 0.90)  P<0.001 |
| Smoking (Current/Former) * | 4,259/18,262 (23.3) | 43,829/134,927 (32.5) | 0.63 (0.61 – 0.66)  P<0.001 |
| *^1^ Unadjusted odds ratios and 95% confidence intervals for association between individual co-variates and susceptibility among those tested for SARS-CoV-2.*  *^2^ Low income classified as zip codes in the 2 lowest pentiles for median household income.*  *^3^ Poverty classified as zip codes where the percentage of residents in poverty is above the national average (11.8%).*  *^4^ High population density classified as zip codes in the 2 highest pentiles for population density.*  ** Denominator denotes number of non-missing values.* | | | |

**Supplementary Table 4.** Socio-demographic, comorbidity, and clinical factors associated with in-hospital mortality among hospitalized COVID-19 patients through December 11^th^, 2020

|  | **Mortality**  *(n = 708)* | **No Mortality**  *(n = 6,540)* | **OR (95% CI) ^1^** |
| --- | --- | --- | --- |
| **Demographic and Social Characteristics – n (%)** | | | |
| Age – mean (SD) | 71.2 (13.7) | 58.2 (17.3) | 1.05 (1.05 – 1.06)  P<0.001 |
| Female (vs. Male) | 310 (43.8) | 3,235 (49.5) | 0.80 (0.68 – 0.93)  P=0.004 |
| Race |  |  |  |
| White | 454 (64.1) | 4,270 (65.3) | *Reference* |
| Black | 159 (22.5) | 1,460 (22.3) | 1.02 (0.84 – 1.24)  P=0.805 |
| Asian | 38 (5.4) | 317 (4.8) | 1.13 (0.78 – 1.58)  P=0.502 |
| Other / Mixed / Not Reported | 57 (8.1 | 493 (7.5) | 1.09 (0.81 – 1.44)  P=0.572 |
| Hispanic (vs. Non-Hispanic) | 227 (32.1) | 2,453 (37.5) | 0.78 (0.66 – 0.93)  P=0.004 |
| Marital Status |  |  |  |
| Single | 136 (19.2) | 1,743 (26.7) | *Reference* |
| Married / Life Partner / Common Law | 372 (52.5) | 3,624 (55.4) | 1.32 (1.07 – 1.62)  P=0.009 |
| Separated / Divorced / Widowed | 182 (25.7) | 997 (15.2) | 2.34 (1.85 – 2.96)  P<0.001 |
| Unavailable | 18 (2.5) | 176 (2.7) | 1.31 (0.76 – 2.14)  P=0.304 |
| Insurance Type |  |  |  |
| Commercial | 95 (13.4) | 2,391 (36.6) | *Reference* |
| Medicare | 306 (43.2) | 2,522 (38.6) | 3.05 (2.42 – 3.89)  P<0.001 |
| Medicaid | 26 (3.7) | 393 (6.0) | 1.67 (1.05 – 2.56)  P=0.025 |
| Self-Pay | 22 (3.1) | 1,102 (16.9) | 0.50 (0.31 – 0.79)  P=0.004 |
| Other | 259 (36.6) | 132 (2.0) | 49.38 (36.99 – 66.51)  P<0.001 |
| Area Deprivation Index (ADI) | 54 (33.5 – 75) | 53 (31 – 74) | 1.00 (1.00 – 1.00)  P=0.324 |
| Zip Code Residence: Low Income *^2^* | 369 (52.6) | 3,525 (54.5) | 0.93 (0.79 – 1.08)  P=0.346 |
| Zip Code Residence: High Poverty *^3^* | 370 (52.6) | 3,362 (51.8) | 1.03 (0.88 – 1.21)  P=0.691 |
| Zip Code Residence: High Pop Density *^4^* | 299 (42.5) | 2,814 (43.4) | 0.97 (0.82 – 1.13)  P=0.659 |
| **Comorbidities and Co-existing Conditions – n (%)** | | | |
| Charlson Comorbidity Index (CCI) Score | 7 (5 – 10) | 3 (1 – 6) | 1.19 (1.17 – 1.21)  P<0.001 |
| BMI – mean (SD) | 30.1 (8.1) | 31.4 (8.2) | 0.98 (0.97 – 0.99)  P<0.001 |
| Preexisting Cognitive Impairment | 139 (19.6) | 573 (8.8) | 2.54 (2.07 – 3.11)  P<0.001 |
| Obesity | 281 (39.7) | 2,547 (38.9) | 1.03 (0.88 – 1.21)  P=0.700 |
| Hypertension | 621 (87.7) | 4,341 (66.4) | 3.62 (2.89 – 4.58)  P<0.001 |
| Smoking (Current/Former) * | 254/652 (39.0) | 1,637/6,172 (26.5) | 1.77 (1.49 – 2.09)  P<0.001 |
| **Vital Signs at Hospital Admission – n (%)** | | | |
| SBP (mmHg) – mean (SD) | 131.2 (17.7) | 130.1 (20.4) | 1.00 (0.99 – 1.00)  P=0.108 |
| DBP (mmHg) – mean (SD) | 68.5 (9.7) | 71.9 (9.1) | 0.96 (0.95 – 0.96)  P<0.001 |
| Respiratory Rate ≥ 24 breath / min | 295 (41.7) | 919 (14.1) | 4.37 (3.70 – 5.15)  P<0.001 |
| Temperature ≥ 38°C | 25 (3.5) | 131 (2.0) | 1.80 (1.14 – 2.73)  P=0.008 |
| Oxygen Saturation < 94% | 296 (41.8) | 1,043 (16.0) | 3.78 (3.21 – 4.44)  P<0.001 |
| **Hospital Complications – n (%)** | | | |
| Pneumonia | 647 (91.4) | 5,010 (76.6) | 3.24 (2.50 – 4.28)  P<0.001 |
| ARDS | 323 (45.6) | 351 (5.4) | 14.79 (12.33 – 17.77)  P<0.001 |
| Bronchitis | 41 (5.8) | 238 (3.6) | 1.63 (1.14 – 2.26)  P=0.005 |
| Lower Respiratory Tract Infection | 7 (1.0) | 113 (1.7) | 0.57 (0.24 – 1.14)  P=0.148 |
| Acute Renal Injury | 553 (78.1) | 1,975 (30.2) | 8.25 (6.87 – 9.96)  P<0.001 |
| Acute Hepatic Injury | 109 (15.4) | 109 (1.7) | 10.74 (8.13 – 14.19)  P<0.001 |
| Cardiomyopathy or CHF | 293 (41.4) | 1,106 (16.9) | 3.47 (2.95 – 4.08)  P<0.001 |
| Hypoxic Respiratory Failure | 453 (64.0) | 3,535 (54.1) | 1.51 (1.29 – 1.78)  P<0.001 |
| **Therapeutics – n (%)** | | | |
| Hydroxychloroquine | 67 (9.5) | 332 (5.1) | 1.95 (1.47 – 2.56)  P<0.001 |
| Ribavirin | 35 (4.9) | 69 (1.1) | 4.88 (3.19 – 7.33)  P<0.001 |
| Azithromycin | 90 (12.7) | 963 (14.7) | 0.84 (0.67 – 1.06)  P=0.149 |
| Lopinavir/Ritonavir | 10 (1.4) | 17 (0.3) | 5.50 (2.42 – 11.86)  P<0.001 |
| Remdesivir | 210 (29.7) | 1,958 (29.9) | 0.99 (0.83 – 1.17)  P=0.878 |
| Tocilizumab | 201 (28.4) | 619 (9.5) | 3.79 (3.15 – 4.55)  P<0.001 |
| Antithrombotic | 363 (51.3) | 2,226 (34.0) | 2.04 (1.74 – 2.38)  P<0.001 |
| Anticoagulants | 662 (93.5) | 5,891 (90.1) | 1.59 (1.18 – 2.19)  P=0.004 |
| Dexamethasone | 440 (62.1) | 3,665 (56.0) | 1.29 (1.10 – 1.51)  P=0.002 |
| **Laboratory Parameters – n (%)** | | | |
| WBC count <4000/μl | 15 (2.1) | 378 (5.8) | 0.35 (0.20 – 0.57)  P<0.001 |
| Lymphocytes < 20% | 662 (93.5) | 4,364 (67.0) | 7.10 (5.31 – 9.75)  P<0.001 |
| Platelet count <150,000/μl | 186 (26.3) | 668 (10.2) | 3.12 (2.59 – 3.75)  P<0.001 |
| B-natriuretic peptide >100 pg/ml, | 388 (60.7) | 1,330 (29.4) | 3.72 (3.13 – 4.41)  P<0.001 |
| Procalcitonin >0.25 ng/ml | 320 (73.4) | 804 (35.4) | 5.04 (4.02 – 6.36)  P<0.001 |
| Troponin ≥ 0.06 ng/ml | 402 (61.8) | 865 (24.5) | 4.98 (4.18 – 5.94)  P<0.001 |
| Aspartate aminotransferase > 40 U/l | 497 (70.5) | 2,838 (44.7) | 2.95 (2.50 – 3.50)  P<0.001 |
| Alanine aminotransferase >40 U/l | 334 (47.6) | 2,612 (41.2) | 1.29 (1.11 – 1.51)  P=0.001 |
| Total Bilirubin ≥ 1.2 mg/dl | 83 (12.0) | 208 (3.5) | 3.71 (2.83 – 4.84)  P<0.001 |
| C-reactive protein >8.2 ng/ml | 644 (99.1) | 5,036 (92.9) | 8.23 (4.01 – 20.86)  P<0.001 |
| Ferritin level > 3000 ng/ml | 97 (14.9) | 221 (4.0) | 4.20 (3.24 – 5.40)  P<0.001 |
| D-dimer > 0.5 ug/ml | 628 (98.9) | 4,398 (81.3) | 20.62 (10.57 – 48.27)  P<0.001 |
| Creatinine > 1.5 mg/dl | 303 (42.8) | 849 (13.2) | 4.90 (4.15 – 5.78)  P<0.001 |
| Venous lactate > 2.2 mmol/l | 332 (51.7) | 766 (17.0) | 5.22 (4.39 – 6.20)  P<0.001 |
| **Hospital Acuity of Care Factors – n (%)** | | | |
| ICU admission | 602 (85.0) | 1,637 (25.0) | 17.01 (13.80 – 21.17)  P<0.001 |
| Invasive Mechanical Ventilation | 490 (69.2) | 698 (10.7) | 18.81 (15.77 – 22.51)  P<0.001 |
| *^1^ Unadjusted odds ratios and 95% confidence intervals for association between individual co-variates and in-hospital mortality among confirmed COVID-19 cases.*  *^2^ Low income classified as zip codes in the 2 lowest pentiles for median household income.*  *^3^ Poverty classified as zip codes where the percentage of residents in poverty is above the national average (11.8%).*  *^4^ High population density classified as zip codes in the 2 highest pentiles for population density.*  * Denominator denotes number of non-missing values.  *SBP: Systolic Blood Pressure, DBP: Diastolic Blood Pressure, ARDS: Acute Respiratory Distress Syndrome, CHF: Congestive Heart Failure* | | | |
